# The Yin-Yang functions of macrophages in metabolic disorders

**DOI:** 10.1093/lifemedi/lnac035

**Published:** 2022-08-30

**Authors:** Juli Bai, Feng Liu

**Affiliations:** National Clinical Research Center for Metabolic Diseases, Metabolic Syndrome Research Center, The Second Xiangya Hospital of Central South University, Changsha 410011, China; Department of Pharmacology, University of Texas Health at San Antonio, San Antonio, TX 78229, USA; National Clinical Research Center for Metabolic Diseases, Metabolic Syndrome Research Center, The Second Xiangya Hospital of Central South University, Changsha 410011, China

**Keywords:** macrophages, NAFLD, diabetes, thermogenesis, dysbiosis

## Abstract

Macrophages are widely distributed in various metabolic tissues/organs and play an essential role in the immune regulation of metabolic homeostasis. Macrophages have two major functions: adaptive defenses against invading pathogens by triggering inflammatory cytokine release and eliminating damaged/dead cells via phagocytosis to constrain inflammation. The pro-inflammatory role of macrophages in insulin resistance and related metabolic diseases is well established, but much less is known about the phagocytotic function of macrophages in metabolism. In this review, we review our current understanding of the ontogeny, tissue distribution, and polarization of macrophages in the context of metabolism. We also discuss the Yin-Yang functions of macrophages in the regulation of energy homeostasis. Third, we summarize the crosstalk between macrophages and gut microbiota. Lastly, we raise several important but remain to be addressed questions with respect to the mechanisms by which macrophages are involved in immune regulation of metabolism.

## Introduction

An organism needs to cope with its environment to function and survival. It is now well established that the dynamic and constant crosstalks between the immune and metabolic systems play a critical role in the maintenance of organisms’ homeostasis. Immune cells, which are able to respond to environmental signals and assume a wide variety of distinct defense functions, are found in key metabolic tissues such as adipose tissue, the liver, and the muscle. Under pathophysiological conditions, these tissue-resident immune cells secrete various cytokines which may promote inflammation and thus lead to metabolic dysregulation. Indeed, both overactivation and loss of function of these cells are associated with various metabolic diseases such as insulin resistance, type 2 diabetes (T2D), nonalcoholic fatty liver diseases (NAFLD), and muscle impairment [1–3], demonstrating a critical role of the immune system in the regulation of metabolism.

The immune system consists of a complex network of specialized cells, tissues, organs proteins, and chemicals that provides defense against infections. Immune cells can be categorized as lymphocytes (T cells, B cells, and NK cells), neutrophils, monocytes, and/or macrophages, as well as all types of white blood cells. In the current review, we focus only on the roles of macrophages in the development and pathogenesis of metabolic diseases. We discuss the ontogeny, tissue distribution, and polarization of macrophages in response to metabolic alterations. We also summarize recent development in our understanding of the pro- and anti-inflammatory functions of macrophages with an emphasis on several metabolic diseases such as obesity, insulin resistance, T2D, and NASH, focusing on the Yin-Yang regulation of macrophages in disease progression and pathogenesis. Lastly, we discuss knowledge gaps in the communication between macrophages and gut microbiota and put forward several important questions with respect to the mechanisms by which macrophages are involved in the immune regulation of metabolism.

## Macrophage ontogeny and tissue distribution

Macrophages, which are evolutionary conserved phagocytes evolved >500 million years ago [4, 5], were first recognized by the Russian scientist Elia Metchnikoff in 1882 as cells capable of phagocytosing foreign particles (for this contribution Elia Metchnikoff received the Noble prize in Physiology and Medicine in 1908) [6]. Macrophages are distributed in various tissues throughout our body, patrolling for and eliminating pathogens and dead cells. In addition to sensing and responding to infectious challenge and physiologic microenvironmental changes, macrophages have a broad spectrum of immune- and nonimmune-related activities such as regulation of tissue development, remodeling, and homeostasis during development and postnatally [7].

Immune cells such as macrophages undergo metabolic adaptation in different tissues to regulate tissue homeostasis and the balance between organ health and disease [8, 9]. Tissue-resident macrophages were historically considered to originate and renew from hematopoietic stem cells (HSCs)-derived circulating blood monocytes [10]. This view has now been challenged with the finding that most adult tissue-resident macrophages are independent of replenishment by monocytes in the steady state, as evidenced by the finding that the Myb-deficient mice, which lack the bone marrow HSC compartment, still develop tissue-resident macrophage populations including Kupffer cells (KCs), microglia, Langerhans cells, lung alveolar, splenic red pulp, and peritoneal macrophages, indicating the possibilities of other origination sources of resident macrophages [11–15]. In fact, these later investigations reveal that there are two lineages of macrophages in mice, one derives from the yolk sac, the major origin for tissue-resident macrophages, and another from the HSC progeny, responsible for replenishing macrophages pools [8, 9, 11, 13].

**Figure 1.**
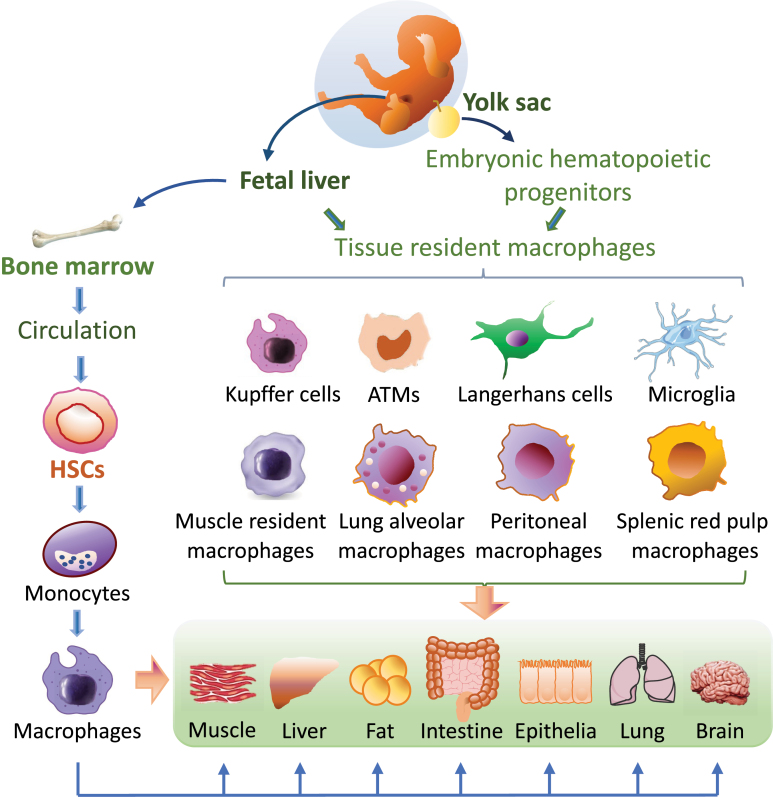
General derivation and distribution of macrophages in the body. Macrophages are derived from two main fetal organs: the fetal liver and yolk sac. Tissue-resident macrophages such as microglia in the central nervous system, Langerhans cells in epidermal tissue, and Kupffer cells in the liver have prenatal origins from the yolk sac and are capable of self-renewal. Hematopoietic stem cells originate from the fetal liver can develop into monocytes and finally be recruited and differentiate into tissue-resident macrophages at inflammatory sites in response to immune and/or metabolic signals. Macrophages resident in adipose tissue and skeletal muscle could be derived from both the yolk sac and the bone marrow.

It is now recognized that tissue-resident macrophages can derive from yolk sac macrophages, fetal liver monocytes, or adult bone-marrow monocytes [16] (Fig. 1). In metabolic organs such as liver, while the yolk-sac-derived tissue-resident macrophages such as KCs are long lived, self-maintaining and only marginally replaced by HSC-derived cells under the steady state conditions [11], there is some evidence showing that KCs could be complemented by recruited monocytes under inflammatory conditions [15, 17]. Consistent with these findings, KC homeostasis is impaired during NASH [18] and the NASH diet induces a partial loss of KC identity and cell death in mice, which could be compensated by gain of adjacent monocyte-derived macrophages [19]. Monocytes could also become self-renewing tissue-resident macrophages in the liver when liver-resident KCs are depleted [20]. It is also interesting to notice that under homeostatic conditions, adipose tissues contain a pool of both yolk-sac-derived and bone-marrow-derived monocytes/macrophages, which coexist and form the “hard wired” heterogenous macrophage population [21, 22]. Skeletal muscle-resident macrophages are also derived from both embryonic hematopoietic progenitors located within the yolk sac and fetal liver as well as definitive HSCs located within the bone marrow of adult mice [23]. In addition to liver, fat, and muscle, high levels of macrophages are also found in the lamina propria of intestine which are specialized to be hyperresponsive to the gut microbiota. The macrophage composition in the intestinal is highly dynamic and there is some evidence showing that the yolk-sac-derived intestinal macrophages are present in the neonatal intestine and do not persist in adult colon, although they are diluted out by HSC-derived macrophages after weaning, a process that continuous throughout adulthood [24]. However, this view was challenged by a fate mapping approach study showing that gut-resident macrophages are populated by both embryonic and monocyte-derived macrophages and the yolk-sac-derived cells persist and self-renew in the specialized intestinal niches in adult [25, 26]. In addition, the subpopulation Tim-4^ + ^CD4^ + ^macrophages are also found to be locally maintained but it is unlikely to be of yolk sac origin [27]. It is interesting to notice that a specific tissue, such as the liver and intestine, may have both embryonically derived- and monocyte-derived macrophages, indicating substantial heterogeneity of the cells which display distinct morphology, specific capability recognizing pathogen, and produce specific inflammatory cytokines. However, it remains to be further clarified as to whether the yolk-sac- and HSC-derived macrophages have similar or different functions and whether these distinct macrophage populations have specialized, organ-specific functions in different tissues. In this review, we only discuss the distribution of macrophages in tissues known to play important roles in metabolism, including fat, liver, muscle, intestine, and brain. Dysregulation of macrophages in other tissues may also contribute to metabolic disorders under certain pathophysiological conditions.

## The pro- and anti-inflammatory functions of macrophages

### Macrophage polarization

Macrophages are functionally plastic cells and sense changes in the surrounding microenvironment. Activation of macrophages by damage- or pathogen-associated molecular patterns (DAMP/PAMPs) in the microenvironment triggers different polarization of the cells to adapt distinct functional phenotypes. Macrophages are originally classified as two distinct subsets, the classically activated (M1) and alternatively activated (M2) macrophages [28]. Macrophage polarization to the M1 phenotype is induced by lipopolysaccharide (LPS), Th1 pro-inflammatory cytokines such as interferon-γ, tumor necrosis factor alpha (TNF-α), and interleukin-1β (IL-1β), and/or small molecules produced during glucose metabolism such as ATP, reactive oxygen species, nitric oxide, and NADPH, whereas M2 phenotype is triggered by Th2 cytokines such as IL-4 and IL-13 as well as anti-inflammatory cytokines such as IL-10 and transforming growth factor beta (TGF-β), or glucocorticoids [29]. M1 macrophages enhance inflammation by secreting pro-inflammatory cytokines such as IL-1β, IL-6, IL-12, IL-23, and TNF-α [30]. M2 macrophages, on the other hand, help control inflammation by producing anti-inflammatory cytokines such as IL-10 and TGF-β [30, 31]. M2 macrophages also suppress inflammation and enhance tissue repair by promoting the differentiation of Th2 cells and Treg cells while reducing pro-inflammatory cytokine release from Th1 cells [32]. However, many recent studies show that the M1/M2 paradigm is not sufficient to embrace all states of macrophage activation. M1 or M2 polarization activation markers can coexist in tissue macrophages [33, 34], which may be expressed simultaneously [35]. Thus, the complex *in vivo* phenotypes and functions of macrophages are likely be determined by distinct upstream signaling stimuli and by specific tissue microenvironments. It should also be pointed out that macrophage polarization is largely established using *ex vivo* modeling systems (such as murine bone-marrow- or human monocyte-derived macrophages) and in response to known polarizing agents, the bioenergetic adaptations of tissue-resident macrophages in responses to various metabolic stimuli and the complex microenvironment *in vivo* remain to be addressed.

### The pro- and anti-inflammatory functions of macrophages in metabolic diseases

As mentioned above, macrophages have two major functions: adaptive defenses by releasing inflammatory cytokines and reducing inflammation by engulfing damaged/dead cells via phagocytosis. In response to specific tissue environmental changes, distinct subsets, and specific polarization states of the macrophages may enhance or resolve inflammation to maintain homeostasis. Interestingly, there is some evidence showing that anti-inflammatory macrophages have higher phagocytic capability [36], which is known to play a central role in innate immunity by eliminating pathogenic bacteria, fungi, and dead or damaged cells. Therefore, activation of macrophages may promote or reduce inflammation, depending on both the state of the macrophages and the nature of the diseases. For example, low-grade chronical inflammation, which could be induced by obesity or other environmental factors, has been found to be a major trigger for metabolic diseases such as insulin resistance, nonalcoholic fatty liver (NAFL), and T2D [37]. For some other disorders such as nonalcoholic steatohepatitis (NASH), cell injury/apoptosis-induced inflammation appears to play a major role in the progression of these diseases [38]. Thus, while suppressing the pro-inflammatory function of macrophages is beneficial for improving insulin resistance, fatty liver, and T2D, suppressing the phagocytic capability of macrophages may be detrimental to cell damage/death-related diseases such as NASH. Further evidence will be needed to verify this possibility.

### Regulation of macrophage functions by metabolites

It is well known that macrophages modulate tissue microenvironment and maintain tissue homeostasis by coordinating other immune cell functions via cytokine signaling [39, 40]. Recent studies show that macrophage metabolites are also key elements to mediate and regulate macrophage functions [39, 41]. Several metabolites have recently been identified in macrophages and one of them is itaconic acid (ITA), an antimicrobial compound that is not generally classified as a mammalian metabolite. Using NMR-based metabolomics and ^13^C-labeling, Strelko *et al.* [42] found that ITA is synthesized from the citric acid cycle intermediate cis-aconitic acid during macrophage activation. Activation of macrophages by LPS and IFN-γ markedly increased ITA production and secretion, which in turn suppresses LPS-induced gene expression in macrophages [43] and alleviates lung and liver injury in mice [43]. Very recently, two naturally occurring isomers of ITA, mesaconate [44], and citraconate [44], have also been identified. All three isomers profoundly alter amino acid metabolism, modulate cytokine/chemokine release, decrease interferon signaling, and reduce oxidative stress [45]. However, these isoforms show some difference in their target specificities. Unlike ITA, which represses tricarboxylic acid cycle activity and cellular respiration by inhibiting Tet methylcytosine dioxygenase 2 and succinate dehydrogenase, mesaconate inhibits only the glycolytic activity [44]. While neither mesaconate nor ITA treatment impairs inflammasome activation [44], citraconate reduces interferon responses and oxidative stress, and modulates inflammation and cell metabolism [45]. Of the three isomers, only citraconate inhibits catalysis of itaconate by the mitochondrial metabolic enzyme cis-aconitate decarboxylase [45]. These studies reveal previously unidentified biosynthetic pathways in cell metabolism and identify novel metabolites that likely play roles in macrophage-based immune response and metabolic regulation.

## The roles of adipose tissue macrophages in the regulation of energy homeostasis

Obesity, which is associated with various metabolic and cardiovascular diseases as well as certain types of cancer, has become one of the most serious public health crises worldwide [46]. Obesity greatly induces macrophage accumulation in mouse adipose tissue, which is increased from ~5% in lean subjects to ~50% of all adipose tissue cells in obese mice [47–49]. A similar obesity-induced increase in adipose tissue macrophage (ATM) population is also observed in humans. The drastic increase in ATMs in adipose tissue suggests a critical role of macrophages in overnutrition-induced dysregulation of energy homeostasis and metabolism.

Obesity is caused by imbalanced nutrient input and energy expenditure, which leads to the storage of the excess nutrients as lipids in adipose tissues and ectopically other metabolically important tissues such as liver and skeletal muscle, leading to various medical consequences such as insulin resistance, coronary heart disease, and T2D [50]. Adipose tissue can be classified into three types: white, brown, and beige adipose tissues, which differ in their structure, location, and functions. The major role of white adipose tissue (wAT) is energy storage while the brown (BAT) and beige adipose tissues are adaptive thermogenic fat which dissipate energy in the form of heat and offers a therapeutic potential to counteract obesity and metabolic disorders. The classic view of BAT thermogenesis is that a centrally processed cold-sensation triggers the release of norepinephrine (NE) from its sympathetic nerve terminals, stimulating β3-adrenoceptor signaling in BAT that upregulate uncoupling protein 1 (UCP1) [51]. However, various evidence indicates that macrophages also play a noncanonical role in modulating the thermogenic function of adipose tissues to regulate systemic energy homeostasis [52–54].

### Inhibition of thermogenesis by macrophages

**Figure 2.**
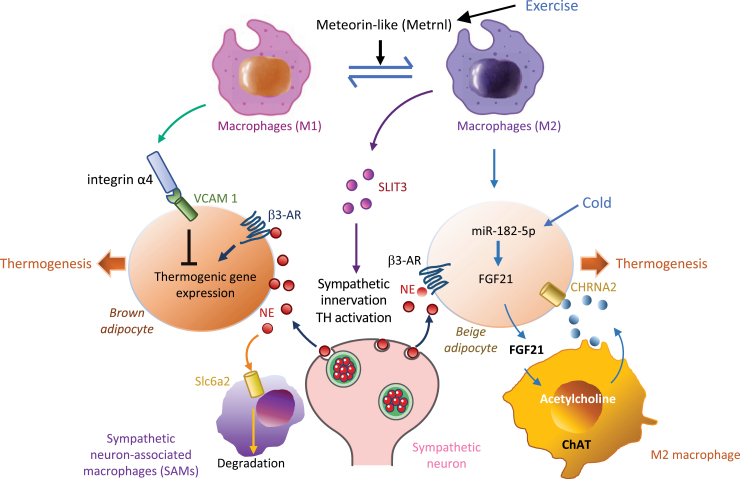
ATM-mediated adaptive thermogenesis. Sympathetic neurons activate brown and beige fat by releasing NEs, which promotes thermogenic gene expression in brown and beige adipocytes by activating β3-AR-mediated signaling pathway. Macrophage subpopulations within thermogenic adipose tissues, including brown and subcutaneous white fat, have distinct roles in regulating energy homeostasis. M1 macrophages inhibit thermogenic gene expression in adjacent adipocytes via integrin α4 and its receptor VCAM-1 (green arrows). Alternatively activated M2 ATMs secrete SLIT3 that promotes sympathetic innervation and TH activation, leading to increased thermogenic gene expression via the β3-AR in brown and beige adipocytes (purple arrows). Alternatively activated macrophages also produce acetylcholine to activate thermogenic responses in brown or beige adipocytes through paracrine mechanisms in a cold environment (blue arrows). SAMs subset may suppress thermogenesis via Slc6a2-mediated import/degradation of NEs (golden arrows). AR, adrenergic receptor; ChAT, choline acetyltransferase; CHRNA2, neuronal acetylcholine receptor subunit alpha 2;SLC6a2, solute carrier family 6 member 2; VCAM1, vascular cell adhesion molecule-1.

Macrophages are accumulated in thermogenic adipose tissues in the state of obesity [55]. Infiltration of classically activated macrophages, which is known to induce inflammation, has been found to suppress UCP1 expression in adipose tissues of HFD-fed C57BL/6 mice, while depletion of macrophages using clodronate liposomes eliminated the suppressive effect [55]. M1 macrophages also inhibit UCP1 expression in adjacent adipocytes via integrin α4 and its counter-receptor VCAM-1-mediated adhesive interaction with adipocytes [56] (Fig. 2). Consistent with an inhibitory role of macrophages in thermogenesis, deficient p38 activation in myeloid cells increases macrophage IL-12 production, leading to inhibition of hepatic FGF21 and reduction of thermogenesis in the brown fat [57]. These findings suggest that infiltration of classically activated (M1) macrophages could cause not only insulin resistance but also reduction of energy expenditure in adipose tissues.

### Promotion of thermogenesis and energy expenditure by alterative-activated macrophages

Unlike the classically activated macrophages, accumulating evidence suggests alternatively activated macrophages promote thermogenesis [53]. Meteorin-like (Metrnl) is a circulating factor that is induced in muscle after exercise and in adipose tissue upon cold exposure. Metrnl promotes alternative activation of ATMs and blocking Metrnl actions significantly attenuates chronic cold exposure-induced alternative macrophage activation and thermogenic gene responses *in vivo* [58] (Fig. 2). A single-cell transcriptomic analysis of stromal cells from inguinal wAT (iwAT) of mice revealed an association between increased M2 macrophages and elevated thermogenesis, and depletion of M2 macrophages abrogated iwAT beiging [59]. The mechanisms by which M2 macrophages enhance thermogenesis remain elusive, but several studies suggest that M2 macrophages could produce and secrete catecholamines that stimulate beige fat thermogenesis [60, 61]. However, a later study from a collaboration of six laboratories showed that alternatively activated macrophages do not synthesize relevant amounts of catecholamines [62]. In addition, hematopoietic deletion of tyrosine hydroxylase (TH), an enzyme required for synthesis of catecholamines such as NE, had no effect on cold thermogenesis in adult mice [62], suggesting the presence of alternative mechanism(s) that promotes beige fat thermogenesis. Consistent with this notion, IL-33 induces UCP1 expression in wAT and the induction is independent of the adaptive immune system, eosinophils, or IL-4 receptor signaling but dependent of Group 2 innate lymphoid cells, which regulate beige fat thermogenesis in part via production of enkephalin peptides [63]. However, alternatively activated macrophages have been found to regulate thermogenesis by secreting Slit guidance ligand 3 (SLIT3) under cold exposure conditions, which induces sympathetic innervation and TH activation by binding to sympathetic neurons, thereby promoting NE synthesis and beige fat development [64]. Increased beige adipocyte thermogenesis is also stimulated by the nicotinic acetylcholine receptor (nAChR) signaling pathway, which is activated by acetylcholine locally produced from M2 macrophages [65]. The mechanism triggering acetylcholine production in macrophages was unknown, but a later study showed that the production and secretion of FGF21 in adipocytes, which is mediated by miR-182-5p, plays a key role in cold exposure-induced acetylcholine production in macrophages, uncovering the miR-182-5p/FGF21/acetylcholine/nAChR axis that mediates the crosstalk between adipocytes and macrophages to promote beige fat thermogenesis [66] (Fig. 2). The involvement of M2 macrophages in regulating thermogenesis is also supported by the finding that adipocyte-specific inducible deletion of the gene encoding cannabinoid receptor type-1, which is associated with an increase in M2 macrophages concomitant with enhanced sympathetic tone in adipose tissue, protects mice from diet-induced obesity and alleviates obesity-induced metabolic phenotypes [67]. M2 macrophage polarization is also induced by IL-25, which increases outgrowth of sympathetic nerves in subcutaneous wAT, contributing to adaptive thermogenesis [68]. However, while these findings suggest that M2 macrophages contribute to adaptive thermogenesis in certain physiological settings, it is well known that macrophages are heterogeneous, and their phenotype and functions are regulated by the surrounding microenvironment. Thus, it is unsurprising that some tissue-resident macrophages may exert a repressive role in regulating energy expenditure. Consistent with this view, Pirzgalska *et al.* [69] show that the sympathetic neuron-associated macrophages (SAMs), which are found in both mice and human and show different neural- and adrenergic-related gene expression profiles compared to other macrophage populations, play a key role in the clearance of intracellular NE. SAMs uptake intracellular NE and degrade it via an NE transporter (Slc6a2) and a degradation enzyme (monoamine oxidase; MAOa) (Fig. 2). These results demonstrate that SAMs promote obesity through noradrenaline clearance, suggesting that SAMs and their molecular machinery are potential therapeutic targets for obesity.

## The role of macrophages in low-grade inflammation, insulin resistance, and T2D

Various studies have clearly demonstrated a role of macrophage dysregulation in excessive inflammation and metabolic diseases [70]. Inflammation is a normal physiological defense response of the body to foreign pathogen invasion. However, excessive inflammatory response may lead to serious metabolic diseases such as insulin resistance and T2D. T2D is now well recognized as a chronic, low-grade inflammatory disease characterized by impaired insulin secretion, insulin resistance, glucose intolerance, and hyperglycemia. The first report on a link between inflammation and insulin resistance back to 1993 when Hotamisligil *et al.* [71] found that obesity increased the expression of pro-inflammatory cytokine TNF-α in adipose tissue. Furthermore, they showed that neutralizing TNF-α significantly increased insulin stimulated glucose uptake in obese rats. To date, numerous studies have consistently shown that reducing inflammation is metabolically protective, which alleviates the development of insulin resistance and T2D [72].

Chronic inflammation in adipose tissue is considered as a crucial risk factor for the development of insulin resistance and T2D in obese individuals. The major source of inflammation in adipose tissue remains to be defined, but adipose tissue is well known to comprise multiple immune cells such as monocytes, macrophages, neutrophils, and T cells, in addition to adipocytes [73]. During chronic inflammation, immune cells such as lymphocytes and macrophages accumulate and infiltrate into metabolic tissues including adipose tissue. In adipose tissue of healthy/lean subjects, alternatively activated M2 macrophages secrete predominately anti-inflammatory cytokines. In contrast, obesity induces macrophage polarization and an M1 phenotypic switch in adipose tissue, leading to increased production of pro-inflammatory cytokines such as TNF-α, IL-6, and IL-1β as well as chemokines MCP-1, CCR2, and CCR5. The increased levels of pro-inflammatory cytokines initiate the recruitment of monocytes and M1 macrophages in adipose tissue, leading to the activation of pro-inflammatory signaling pathways such as JNK, ERK, p38, IκB, and IKKβ that inhibit insulin signaling [73–75]. In addition to these signaling pathways, the mammalian target of rapamycin complex 1 (mTORC1) signaling pathway has also been shown to play a key role in obesity-induced macrophage polarization and insulin resistance. In obesity, nutrient sensing by mTORC1 switches ATMs from M2 to M1 [76]. Consistently, activation of the mTORC1 signaling pathway by deleting TSC1 in macrophages intensifies the M1 [77] but suppresses M2 polarization [78]. In agreement with these results, depletion of mTORC1 in macrophages protects mice against HFD-induced adipose tissue inflammation and insulin resistance [79]. Another important regulator of macrophage polarity is the ER stress protein CHOP. CHOP expression is upregulated in adipocytes of HFD-fed mice and CHOP deficiency promotes M2 macrophage polarization, concurrently with alleviated insulin resistance and glucose intolerance [80]. Bone marrow transplantation experiments showed the polarity of ATMs is mainly determined by CHOP [80]. Consistent with the view that ER stress plays a key role in regulating macrophage polarization in adipose tissue, myeloid-specific abrogation of inositol-requiring enzyme 1α promotes M2 macrophage polarization in wAT, greatly increased brown and beige fat development and energy expenditure, and blocked HFD-induced metabolic disorders [81]. These results reveal novel molecular mechanisms regulating macrophage polarization and a link between obesity, macrophage polarization, and metabolic diseases such as insulin resistance.

In addition to triggering insulin resistance in adipose tissues, macrophage polarization has also been found to modulate the function of β cells [82], the cells in the pancreas that produce and release insulin in response to elevated blood glucose levels. Macrophages are in close contact with both β cells and vasculature in mice [83] and may directly provoke or enhance insulin secretion through production of factors such as retinoic acid [84]. Although there are some data suggesting that islet macrophages do not follow the M2 protection vs. M1 deterioration polarization paradigm [85], Yin *et al.* [86] found that infusion of human umbilical cord-derived mesenchymal stem cells, which are known to protect islet function in T2D individuals, promotes macrophage polarization toward an anti-inflammatory M2-like state, correlating with a significant increase in islets recovery in type 2 diabetic mice. Macrophages have also been found to regulate the adaptation of β cells to early weight gain by licensing β cell mass expansion and the required angiogenesis during the early weeks of high-fat diet feeding [87]. Indeed, depleting islet macrophages reduced VEGF-A secretion in both human and mouse islets *ex vivo*, leading to a significant and consistent compromised islet remodeling in terms of size, vascular density, and insulin secretion capacity [87].

## The Yin-Yang function of macrophages in NASH progression

NAFLD, which includes NAFL and the more severe NASH and cirrhosis, has now become the leading cause of end-stage liver failure worldwide [88, 89]. Both resident (KCs) and recruited macrophages have been recognized as key contributors to the development and pathogenesis of NAFLD [90].

NAFL is defined as hepatic steatosis with no evidence of hepatocellular injury and fibrosis. NASH, on the other hand, is characterized by toxic accumulation of fat in the liver (steatosis) that induces inflammation, hepatocellular injury, and varying degrees of fibrosis [38]. The transition from the largely benign hepatic steatosis to NASH is of great clinical importance, given that the latter can progress to end-stage liver diseases such as cirrhosis, liver failure, or hepatocellular carcinoma [38] and affects <100 million people worldwide [91]. Unfortunately, no NASH drug has been approved by any leading regulatory agencies as of today, highlighting the need for better understanding the biological basis and drug targets of NASH. While numerous studies have demonstrated a strong association between NASH and obesity, dyslipidemia, and T2D [38], key pathogenic drivers of NASH initiation and progression remain to be characterized. Liver is a heterogenous tissue composed of multiple cells including hepatocytes and non-parenchymal cells such as liver endothelial cells, hepatic stellate cells, biliary cells, and immune cells such as lymphocytes (T cells and B cells) and macrophages. Accumulating evidence suggests that immune cell activation could be one of the major triggers of NASH instigation and development [92].

**Figure 3.**
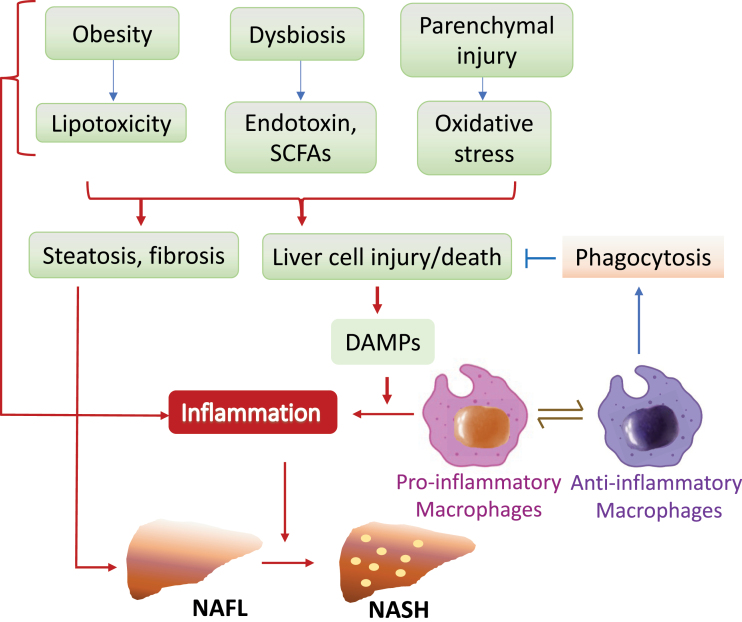
The Yin-Yang functions of macrophages in the development of NASH. Obesity, dysbiosis, and parenchymal injury lead to increased lipotoxicity, endotoxin, and oxidative stress, which induce steatosis, fibrosis, and cell injury/death in the liver. Damaged and deceased cells promote the release of DAMPs, which in turn enhances inflammation in NASH. Increased phagocytosis of macrophages is critical to remove the injured/deceased cells and thus reduces inflammation in NASH.

Among immune cells, macrophage is emerging-studied in the regulation of NASH pathogenesis in recent years. Liver macrophages mainly consist of liver-resident phagocytes (KCs) and bone-marrow-derived recruited monocytes. Both resident and recruited macrophages predominate some common functional abilities such as phagocytosis, recognition of danger signals, cytokine release, and antigen processing, as well as orchestrating immune responses. However, these cells also display some preferences in terms of regulation of tissue homeostasis and responses to acute or chronic injury [93]. In the healthy liver, KCs exist within the hepatic sinusoids where they scavenge bacteria and microbial products from the intestine while mature monocytes show a patrolling behavior. Under the pathophysiological condition, liver macrophages are activated by various factors such as gut-derived endotoxins, lipids and lipid metabolites, and damage-associated molecular patterns (DAMPs), and activation of liver macrophages are associated with enhanced inflammation [90]. Extensive experimental and clinical data suggest that KCs and recruited macrophages are found to be critical in initiating liver damage thus the progression of NASH [90, 94]. A widely accepted view on NASH development is that overaccumulated lipid toxicity causes hepatocyte injury, which release DAMPs that stimulate KCs in the liver. The activated resident KCs then release pro-inflammatory cytokines and chemokines, leading to the consecutive recruitment of inflammatory monocyte-derived macrophages and elevated inflammation that accelerates the progression of NAFL to NASH and cirrhosis [95] (Fig. 3). In line with the view that KCs play a key role in obesity-induced metabolic abnormalities, depletion of liver KCs by administration of gadolinium chloride in mice prevents diet-induced hepatic steatosis and insulin resistance [96] as well as the development of NASH [94].

Interestingly, some recent studies reveal that in addition to sustained hepatic inflammation from immune cells, increased cell damage or death, which derives the production of endogenous danger signals such as alarmins and DAMPs, play key contributing roles in the transition from NAFL to NASH [97–100] (Fig. 3). Consistent with a critical role of cell death in NASH pathogenesis, enhanced hepatocyte apoptosis contributes to early signaling event in diet-induced NASH in mice [101, 102]. Cell death may be accidental or regulated, and regulated cell death (RCD) may either be programmed (occur in the absence of any exogenous environmental perturbation) or may originate from intense or prolonged perturbations of the intracellular or extracellular microenvironment [103]. Beside apoptosis, several types of the RCD such as necroptosis [104], pyroptosis [105], and ferroptosis [106–108] have also been implicated in animal models of NASH and in liver biopsies of patients with NASH [109, 110]. Knockout of the receptor for TNF-related apoptosis-inducing ligand protects mice from HFD-induced macrophage inflammatory responses and high fat, high fructose, and high cholesterol diet-induced hepatocyte apoptosis and NASH, revealing a link between death receptor-induced inflammation and NASH [101]. Since enhanced cell death contributes to early signaling event in NASH pathogenesis, the clearance of damaged or dead cells might be critical to counteract NASH progression. Indeed, selective depletion of phagocytic macrophage in mice displays accumulated dead cells in liver and are more suspectable to NASH [97]. These findings demonstrate that efficient efferocytosis of dead cells by liver macrophages is essential for protecting against NASH [97, 111]. Thus, macrophages may act as a double-edged sword in NASH progression given that they may promote inflammation if overactivated but may suppress inflammation by rapidly removing damaged and dead cells via phagocytosis [112, 113]. However, how these innate immune responses are initiated and coordinately regulated in response to nutritional and local environmental cues-induced NASH development remain to be further elucidated.

## The roles of macrophages in dysbiosis-induced metabolic diseases

Gut microbiota is an integral part of the human body and disrupting microbiota homeostasis (dysbiosis) has been implicated in inducing low-grade inflammation that activates tissue-resident macrophages, contributing to a variety of metabolic diseases such as diabetes, obesity, and metabolic syndrome [23, 29]. The precise mechanisms by which gut microbiota dysbiosis induces metabolic diseases remain to be further elucidated, but numerous studies suggests that macrophages are one of the key players in mediating the interaction between gut microbiota and host tissues. For example, treating specific pathogen-free mice with the antibiotic vancomycin led to a significant increase in body weight, cecum weight, and the gastrointestinal transit time, concurrently with a significant increase in M1 and a significant decrease in M2 macrophages in the mucosal and muscular layers of the colon of the mice [114]. Early and frequent uncontrolled use of antibiotics have also shown to disrupt the microbiome and increase the risk of overweight or obesity in both childhood and adults [115, 116]. Inhibition of butyrate production by antibiotics promotes the pro-inflammatory polarization of the intestinal macrophages, leading to a global dysfunction of the immune response [117]. Interestingly, a recent study shows that unhealthy lifestyle habits such as low vegetable intake, high processed meat consumption, and sedentary lifestyle trigger accumulation of pro-inflammatory intestinal macrophages, potentially via recruited blood monocytes, in the stomach, duodenum, and colon of obese human individuals [118]. M1 macrophage polarization in the intestine has been shown to inhibit gastrointestinal motility [114] and compromise barrier function, which consequently causes the leakage of gut microbiota-derived microbial factors or LPS, triggering inflammasome in colonic macrophages to induce sustain intestinal inflammation [119, 120]. Consistent with the view that dysregulation of intestinal macrophages contributes to metabolic dysfunction, there is strong evidence that the innate and adaptive responses in the gut affect the maintenance of the intestinal barrier, systemic inflammation, and glucose metabolism, whose dysregulation contributes to the pathophysiology of obesity and T2D [121, 122].

In addition to be regulated by signals from the host, a large amount of evidence showing that the polarization of macrophages could also be regulated by metabolites produced from microbiota. Under healthy conditions, gut microbiota produces various metabolites such as short-chain fatty acids (SCFAs), butyrate, bile acids, and tryptophan metabolites that are essential for glycolysis, the Krebs cycle, oxidative phosphorylation, and amino acid and fatty acid metabolism of the host cells including metabolic relevant cells such as adipocytes, hepatocytes, β cells as well as immune cells such as macrophages [29, 123, 124]. Transferring the microbiota from healthy donors has been found to improve the body weight and glycemia in both mouse [125] and human [126] obese and diabetic receivers. In addition, targeting gut microbiota dysbiosis by a variety of approaches such as the treatment with probiotics and prebiotics, and the utilization of microbiota-derived SCFAs and butyrate all show beneficial effects on metabolic disorders, including reprogramming macrophage metabolism toward an anti-inflammatory M2 phenotype [29, 117] and enhancing the phagocytic and antimicrobial activities [127]. Besides SCFAs and butyrate, a recent study shows that upregulating microbiota-derived polyamines such as putrescine and spermidine, increases the abundance of M2 macrophages in the colon and promotes longevity in mice [128]. Interestingly, helminth parasite infection-induced M2 macrophage polarization alleviates HFD-induced obesity in mice [129]. Adoptive transfer of macrophages from helminth-infected wT mice conferred protection against HFD-induced obesity in the recipients [129]. The precise underlying mechanism remains to be established but *Heligmosomoides polygyrus* infection has been shown to induce a significant change in the composition of the gut microbiota in mice [130, 131], suggesting that a potential link between gut microbiota and intestinal function in regulating energy homeostasis.

Dysregulation of macrophage function has also been found to be associated with maternal obesity-induced metabolic disorders. Maternal obesity is associated with increased risk for offspring obesity and NAFLD [132–135], but the causal drivers of this association are unclear. By comparing germ-free mice colonized with stool microbes from 2-week-old infants born to obese (Inf-Ob) or normal-weight (Inf-Nw) mothers, Soderborg *et al.* [136] showed that the germ-free mice colonized with stool microbes from Inf-Ob mice displayed increased endoplasmic reticulum stress and innate immunity together with histological signs of pediatric cases of NAFLD compared to mice colonized with stool microbes from Inf-Nw mice. Treating the germ-free mice with stool microbes from the Inf-Ob mice also show increased intestinal permeability, reduced macrophage phagocytosis, and dampened cytokine production suggestive of impaired macrophage function. These results highlight a critical role of macrophages in maternal obesity-associated infant dysbiosis in childhood obesity and NAFLD. Taken together, these findings demonstrate an important role of macrophages in dysbiosis-induced metabolic diseases.

**Figure 4.**
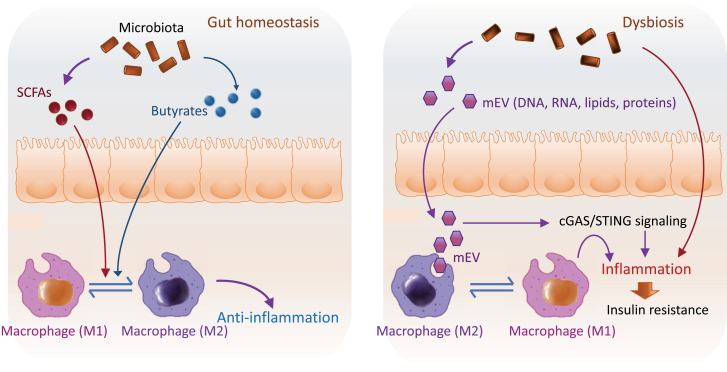
Macrophages dysregulation mediates gut microbiota dysbiosis-induced metabolic diseases. Under healthy conditions, gut microbiota produces various metabolites such as SCFAs, butyrate, and other metabolites that are essential for the host cell metabolism (left). Under obesity conditions, gut microbial DNA-containing mEVs transport a variety of molecules such as RNA, DNA, lipids, and proteins to metabolic tissues where they activate macrophages and the cGAS/STING signaling pathway, triggering inflammation, and insulin resistance.

Another beneficial function of macrophages is to alleviate inflammation and insulin resistance by clearing microbiota-derived products from the bloodstream in the context of obesity. Under obesity conditions, gut microbial DNA-containing extracellular vesicles (mEVs), which serve as vehicles to transport a variety of molecules such as RNA, DNA, lipids, and proteins between the neighbor or distant cells [137], can reach metabolic tissues where they induce inflammation and insulin resistance [138]. Depletion of DRIg (complement receptor of the immunoglobulin superfamily) in macrophages results in the spread of mEVs into distant metabolic tissues, subsequently exacerbating tissue inflammation and metabolic disorders [139]. The initiation of tissue inflammation is likely mediated by microbial DNA-induced activation of the cGAS/STING pathway, a key DNA sensor that plays a critical role in regulating metabolic homeostasis [140, 141], given that depletion of microbial DNA blunts the pathogenic effects of mEVs and deletion of cGAS prevented the suppressive effect of obese mEVs on insulin action [139] (Fig. 4). These results demonstrate that macrophage deficiency may contribute to the development of obesity-associated tissue inflammation and metabolic diseases, revealing again a Yin-Yang function of macrophages in inflammation and metabolic disorders. Thus, a better mechanistic understanding of the roles of macrophages in the content of metabolic disease stages, tissue microenvironment, and their communication partners would be of great importance in developing appropriate therapeutic approaches for the prevention and treatment of metabolic diseases.

## Perspectives and future directions

In this review, we summarize current understanding of the roles of macrophages in the regulation of metabolism. Macrophages are functionally plastic cells, distributed into various tissues, and are highly sensitive to changes in their surrounding microenvironment. The two major functions of macrophages are inflammatory responses against pathogen invasion and phagocytosis to remove damaged/diseased cells to constrict inflammation in a timely manner. However, while the immunomodulation feature of macrophages is well recognized as a key player in various metabolic diseases such as insulin resistance and T2D, the role of the phagocytotic function of macrophages in the development and pathogenesis of metabolic disorders remains to be further defined. Several important questions with respect to the effect of macrophage dysregulation in metabolic diseases and their mechanisms of action remain to be addressed. First, it remains unclear as to under what conditions the pro- or anti-inflammatory function of a macrophage comes into play to promote or alleviate a metabolic disease. An answer to this question is important given that the immunomodulation and phagocytic functions of macrophages could have opposite effects on the development and pathogenesis of a disease. Second, whether the Yin or Yang function of macrophages is specifically induced by a unique signal, by the disease microenvironment orchestrated by multisignals, or by the nature and/or the stages of a disease? Third, what are the key signaling pathways regulating the phagocytic capability to rapidly remove damaged/deceased cells to alleviate the inflammatory phenotypes of a metabolic disease-induced mainly by cell injury/death? Fourth, how do macrophages communicate with other resident cell populations in a metabolic tissue to promote or reduce inflammation? Specifically, do these communications involve the active secretion of molecules from macrophages that impact adjacent cell populations, or regulated by signals from the neighboring cells that induce the Yin or Yang function of a macrophage? In addition to their potential involvement in metabolic disorders, macrophages have also been found to be an excellent target for nanomedicine. Nanoparticles (NPs) have now been widely used for drug delivery in disease treatment due to their stability, biocompatibility, blood circulation, immunogenicity, and capability to control drug release [142, 143]. Due to the phagocytic nature, monocytes, and macrophages are well recognized as nanomedicinal targets for NP-mediated drug delivery [144, 145]. A better mechanistic understanding of macrophages’ function and regulation of macrophages should provide new insights into the challenges and opportunities for developing/optimizing novel therapeutic diagnosis or treatment of metabolic diseases such as obesity, T2D and NASH and other diseases as well.

## References

[1] Buck MD, Sowell RT, Kaech SM, et al. Metabolic instruction of immunity. Cell 2017;169:570–86. 28475890 10.1016/j.cell.2017.04.004 PMC5648021

[2] Cinkajzlova A, Mraz M, Haluzik M. Adipose tissue immune cells in obesity, type 2 diabetes mellitus and cardiovascular diseases. J Endocrinol 2021;252:R1–R22. 34592714 10.1530/JOE-21-0159

[3] Lin SZ, Fan JG. Peripheral immune cells in NAFLD patients: a spyhole to disease progression. EBioMedicine 2022;75:103768. 34929490 10.1016/j.ebiom.2021.103768 PMC8693289

[4] Cooper MD, Alder MN. The evolution of adaptive immune systems. Cell 2006;124:815–22. 16497590 10.1016/j.cell.2006.02.001

[5] Hirano M, Das S, Guo P, et al. The evolution of adaptive immunity in vertebrates. Adv Immunol 2011;109:125–57. 21569914 10.1016/B978-0-12-387664-5.00004-2

[6] Gordon S. Phagocytosis: the legacy of metchnikoff. Cell 2016b;166:1065–8. 27565334 10.1016/j.cell.2016.08.017

[7] Gordon S, Pluddemann A. Tissue macrophages: heterogeneity and functions. BMC Biol 2017;15:53. 28662662 10.1186/s12915-017-0392-4 PMC5492929

[8] Caputa G, Castoldi A, Pearce EJ. Metabolic adaptations of tissue-resident immune cells. Nat Immunol 2019;20:793–801. 31213715 10.1038/s41590-019-0407-0

[9] Nobs SP, Kopf M. Tissue-resident macrophages: guardians of organ homeostasis. Trends Immunol 2021;42:495–507. 33972166 10.1016/j.it.2021.04.007

[10] van Furth R, Cohn ZA. The origin and kinetics of mononuclear phagocytes. J Exp Med 1968;128:415–35. 5666958 10.1084/jem.128.3.415 PMC2138527

[11] Gomez Perdiguero E, Klapproth K, Schulz C, et al. Tissue-resident macrophages originate from yolk-sac-derived erythro-myeloid progenitors. Nature 2015;518:547–51. 25470051 10.1038/nature13989 PMC5997177

[12] Hashimoto D, Chow A, Noizat C, et al. Tissue-resident macrophages self-maintain locally throughout adult life with minimal contribution from circulating monocytes. Immunity 2013;38:792–804. 23601688 10.1016/j.immuni.2013.04.004 PMC3853406

[13] Schulz C, Gomez Perdiguero E, Chorro L, et al. A lineage of myeloid cells independent of Myb and hematopoietic stem cells. Science 2012;336:86–90. 22442384 10.1126/science.1219179

[14] Watanabe S, Alexander M, Misharin AV, et al. The role of macrophages in the resolution of inflammation. J Clin Invest 2019;129:2619–28. 31107246 10.1172/JCI124615 PMC6597225

[15] Yona S, Kim KW, Wolf Y, et al. Fate mapping reveals origins and dynamics of monocytes and tissue macrophages under homeostasis. Immunity 2013;38:79–91. 23273845 10.1016/j.immuni.2012.12.001 PMC3908543

[16] van de Laar L, Saelens W, De Prijck S, et al. Yolk sac macrophages, fetal liver, and adult monocytes can colonize an empty niche and develop into functional tissue-resident macrophages. Immunity 2016;44:755–68. 26992565 10.1016/j.immuni.2016.02.017

[17] Beattie L, Sawtell A, Mann J, et al. Bone marrow-derived and resident liver macrophages display unique transcriptomic signatures but similar biological functions. J Hepatol 2016;65:758–68. 27262757 10.1016/j.jhep.2016.05.037 PMC5028381

[18] Tran S, Baba I, Poupel L, et al. Impaired kupffer cell self-renewal alters the liver response to lipid overload during non-alcoholic steatohepatitis. Immunity 2020;53:627–40.e625. 32562600 10.1016/j.immuni.2020.06.003

[19] Seidman JS, Troutman TD, Sakai M, et al. Niche-specific reprogramming of epigenetic landscapes drives myeloid cell diversity in nonalcoholic steatohepatitis. Immunity 2020;52:1057–74.e7 e1057. 32362324 10.1016/j.immuni.2020.04.001 PMC7305990

[20] Scott CL, Zheng F, De Baetselier P, et al. Bone marrow-derived monocytes give rise to self-renewing and fully differentiated Kupffer cells. Nat Commun 2016;7:10321. 26813785 10.1038/ncomms10321 PMC4737801

[21] Cox N, Geissmann F. Macrophage ontogeny in the control of adipose tissue biology. Curr Opin Immunol 2020;62:1–8. 31670115 10.1016/j.coi.2019.08.002 PMC7067643

[22] Hassnain Waqas SF, Noble A, Hoang AC, et al. Adipose tissue macrophages develop from bone marrow-independent progenitors in Xenopus laevis and mouse. J Leukoc Biol 2017;102:845–55. 28642277 10.1189/jlb.1A0317-082RR PMC5574031

[23] Wang J, Chen WD, Wang YD. The relationship between gut microbiota and inflammatory diseases: the role of macrophages. Front Microbiol 2020;11:1065. 32582063 10.3389/fmicb.2020.01065 PMC7296120

[24] Bain CC, Bravo-Blas A, Scott CL, et al. Constant replenishment from circulating monocytes maintains the macrophage pool in the intestine of adult mice. Nat Immunol 2014;15:929–37. 25151491 10.1038/ni.2967 PMC4169290

[25] De Schepper S, Verheijden S, Aguilera-Lizarraga J, et al. Self-maintaining gut macrophages are essential for intestinal homeostasis. Cell 2018;175:400–15 e413. 30173915 10.1016/j.cell.2018.07.048

[26] Ide S, Yahara Y, Kobayashi Y, et al. Yolk-sac-derived macrophages progressively expand in the mouse kidney with age. Elife 2020;9:1. 10.7554/eLife.51756 PMC7205460 32301704

[27] Shaw TN, Houston SA, Wemyss K, et al. Tissue-resident macrophages in the intestine are long lived and defined by Tim-4 and CD4 expression. J Exp Med 2018;215:1507–18. 29789388 10.1084/jem.20180019 PMC5987925

[28] Mills CD, Kincaid K, Alt JM, et al. M-1/M-2 macrophages and the Th1/Th2 paradigm. J Immunol 2000;164:6166–73. 10843666

[29] Belizario JE, Faintuch J, Garay-Malpartida M. Gut microbiome dysbiosis and immunometabolism: new frontiers for treatment of metabolic diseases. Mediators Inflamm 2018;2018:2037838. 30622429 10.1155/2018/2037838 PMC6304917

[30] Italiani P, Boraschi D. From monocytes to M1/M2 macrophages: phenotypical vs. functional differentiation. Front Immunol 2014;5:514. 25368618 10.3389/fimmu.2014.00514 PMC4201108

[31] Arora S, Dev K, Agarwal B, et al. Macrophages: their role, activation and polarization in pulmonary diseases. Immunobiology 2018;223:383–96. 29146235 10.1016/j.imbio.2017.11.001 PMC7114886

[32] Chu F, Shi M, Zheng C, et al. The roles of macrophages and microglia in multiple sclerosis and experimental autoimmune encephalomyelitis. J Neuroimmunol 2018;318:1–7. 29606295 10.1016/j.jneuroim.2018.02.015

[33] Orecchioni M, Ghosheh Y, Pramod AB, et al. Macrophage polarization: different gene signatures in M1(LPS+) vs. classically and M2(LPS-) vs. alternatively activated macrophages. Front Immunol 2019;10:1084. 31178859 10.3389/fimmu.2019.01084 PMC6543837

[34] Trombetta AC, Soldano S, Contini P, et al. A circulating cell population showing both M1 and M2 monocyte/macrophage surface markers characterizes systemic sclerosis patients with lung involvement. Respir Res 2018;19:186. 30249259 10.1186/s12931-018-0891-z PMC6154930

[35] Novak ML, Koh TJ. Macrophage phenotypes during tissue repair. J Leukoc Biol 2013;93:875–81. 23505314 10.1189/jlb.1012512 PMC3656331

[36] Jaggi U, Yang M, Matundan HH, et al. Increased phagocytosis in the presence of enhanced M2-like macrophage responses correlates with increased primary and latent HSV-1 infection. PLoS Pathog 2020;16:e1008971. 33031415 10.1371/journal.ppat.1008971 PMC7575112

[37] McNelis JC, Olefsky JM. Macrophages, immunity, and metabolic disease. Immunity 2014;41:36–48. 25035952 10.1016/j.immuni.2014.05.010

[38] Sheka AC, Adeyi O, Thompson J, et al. Nonalcoholic steatohepatitis: a review. JAMA 2020;323:1175–83. 32207804 10.1001/jama.2020.2298

[39] Geeraerts X, Bolli E, Fendt SM, et al. Macrophage metabolism as therapeutic target for cancer, atherosclerosis, and obesity. Front Immunol 2017;8:289. 28360914 10.3389/fimmu.2017.00289 PMC5350105

[40] Kim KW, Zhang N, Choi K, et al. Homegrown macrophages. Immunity 2016;45:468–70. 27653599 10.1016/j.immuni.2016.09.006

[41] Artyomov MN, Sergushichev A, Schilling JD. Integrating immunometabolism and macrophage diversity. Semin Immunol 2016;28:417–24. 27771140 10.1016/j.smim.2016.10.004 PMC5333784

[42] Strelko CL, Lu W, Dufort FJ, et al. Itaconic acid is a mammalian metabolite induced during macrophage activation. J Am Chem Soc 2011;133:16386–9. 21919507 10.1021/ja2070889 PMC3216473

[43] Chen LL, Morcelle C, Cheng ZL, et al. Itaconate inhibits TET DNA dioxygenases to dampen inflammatory responses. Nat Cell Biol 2022b;24:353–63. 35256775 10.1038/s41556-022-00853-8 PMC9305987

[44] He W, Henne A, Lauterbach M, et al. Mesaconate is synthesized from itaconate and exerts immunomodulatory effects in macrophages. Nat Metab 2022;4:524–33. 35655024 10.1038/s42255-022-00565-1 PMC9744384

[45] Chen F, Elgaher WAM, winterhoff M, et al. Citraconate inhibits ACOD1 (IRG1) catalysis, reduces interferon responses and oxidative stress, and modulates inflammation and cell metabolism. Nat Metab 2022a;4:534–46. 35655026 10.1038/s42255-022-00577-x PMC9170585

[46] Hruby A, Hu FB. The epidemiology of obesity: a big picture. PharmacoEcon 2015;33:673–89. 10.1007/s40273-014-0243-x PMC4859313 25471927

[47] Boutens L, Stienstra R. Adipose tissue macrophages: going off track during obesity. Diabetologia 2016;59:879–94. 26940592 10.1007/s00125-016-3904-9 PMC4826424

[48] Weisberg SP, McCann D, Desai M, et al. Obesity is associated with macrophage accumulation in adipose tissue. J Clin Invest 2003;112:1796–808. 14679176 10.1172/JCI19246 PMC296995

[49] Harman-Boehm I, Bluher M, Redel H, et al. Macrophage infiltration into omental versus subcutaneous fat across different populations: effect of regional adiposity and the comorbidities of obesity. J Clin Endocrinol Metab 2007;92:2240–7. 17374712 10.1210/jc.2006-1811

[50] Smith U, Kahn BB. Adipose tissue regulates insulin sensitivity: role of adipogenesis, de novo lipogenesis and novel lipids. J Intern Med 2016;280:465–75. 27699898 10.1111/joim.12540 PMC5218584

[51] Saito M, Matsushita M, Yoneshiro T, et al. Brown adipose tissue, diet-induced thermogenesis, and thermogenic food ingredients: from mice to men. Front Endocrinol (Lausanne) 2020;11:222. 32373072 10.3389/fendo.2020.00222 PMC7186310

[52] Qiu Y, Shan B, Yang L, et al. Adipose tissue macrophage in immune regulation of metabolism. Sci China Life Sci 2016;59:1232–40. 27837402 10.1007/s11427-016-0155-1

[53] Rahman MS, Jun H. The adipose tissue macrophages central to adaptive thermoregulation. Front Immunol 2022;13:884126. 35493493 10.3389/fimmu.2022.884126 PMC9039244

[54] Russo L, Lumeng CN. Properties and functions of adipose tissue macrophages in obesity. Immunology 2018;155:407–17. 30229891 10.1111/imm.13002 PMC6230999

[55] Sakamoto T, Nitta T, Maruno K, et al. Macrophage infiltration into obese adipose tissues suppresses the induction of UCP1 level in mice. Am J Physiol Endocrinol Metab 2016;310:E676–87. 26884382 10.1152/ajpendo.00028.2015

[56] Chung KJ, Chatzigeorgiou A, Economopoulou M, et al. A self-sustained loop of inflammation-driven inhibition of beige adipogenesis in obesity. Nat Immunol 2017;18:654–64. 28414311 10.1038/ni.3728 PMC5436941

[57] Crespo M, Nikolic I, Mora A, et al. Myeloid p38 activation maintains macrophage-liver crosstalk and BAT thermogenesis through IL-12-FGF21 axis. Hepatology 2022. doi: 10.1002/hep.32581. PMC9936978 35592906

[58] Rao RR, Long JZ, White JP, et al. Meteorin-like is a hormone that regulates immune-adipose interactions to increase beige fat thermogenesis. Cell 2014;157:1279–91. 24906147 10.1016/j.cell.2014.03.065 PMC4131287

[59] Henriques F, Bedard AH, Guilherme A, et al. Single-cell RNA profiling reveals adipocyte to macrophage signaling sufficient to enhance thermogenesis. Cell Rep 2020;32:107998. 32755590 10.1016/j.celrep.2020.107998 PMC7433376

[60] Nguyen KD, Qiu Y, Cui X, et al. Alternatively activated macrophages produce catecholamines to sustain adaptive thermogenesis. Nature 2011;480:104–8. 22101429 10.1038/nature10653 PMC3371761

[61] Qiu Y, Nguyen KD, Odegaard JI, et al. Eosinophils and type 2 cytokine signaling in macrophages orchestrate development of functional beige fat. Cell 2014;157:1292–308. 24906148 10.1016/j.cell.2014.03.066 PMC4129510

[62] Fischer K, Ruiz HH, Jhun K, et al. Alternatively activated macrophages do not synthesize catecholamines or contribute to adipose tissue adaptive thermogenesis. Nat Med 2017;23:623–30. 28414329 10.1038/nm.4316 PMC5420449

[63] Brestoff JR, Kim BS, Saenz SA, et al. Group 2 innate lymphoid cells promote beiging of white adipose tissue and limit obesity. Nature 2015;519:242–6. 25533952 10.1038/nature14115 PMC4447235

[64] Wang YN, Tang Y, He Z, et al. Slit3 secreted from M2-like macrophages increases sympathetic activity and thermogenesis in adipose tissue. Nat Metab 2021;3:1536–51. 34782792 10.1038/s42255-021-00482-9

[65] Jun H, Yu H, Gong J, et al. An immune-beige adipocyte communication via nicotinic acetylcholine receptor signaling. Nat Med 2018;24:814–22. 29785025 10.1038/s41591-018-0032-8 PMC5992032

[66] Meng, W, Xiao T, Liang X, et al. The miR-182-5p/FGF21/acetylcholine axis mediates the crosstalk between adipocytes and macrophages to promote beige fat thermogenesis. JCI Insight 2021;6:e150249. 34264867 10.1172/jci.insight.150249 PMC8492300

[67] Ruiz de Azua I, Mancini G, Srivastava RK, et al. Adipocyte cannabinoid receptor CB1 regulates energy homeostasis and alternatively activated macrophages. J Clin Invest 2017;127:4148–62. 29035280 10.1172/JCI83626 PMC5663356

[68] Li L, Ma L, Zhao Z, et al. IL-25-induced shifts in macrophage polarization promote development of beige fat and improve metabolic homeostasis in mice. PLoS Biol 2021;19:e3001348. 34351905 10.1371/journal.pbio.3001348 PMC8341513

[69] Pirzgalska RM, Seixas E, Seidman JS, et al. Sympathetic neuron-associated macrophages contribute to obesity by importing and metabolizing norepinephrine. Nat Med 2017;23:1309–18. 29035364 10.1038/nm.4422 PMC7104364

[70] Chakarov S, Bleriot C, Ginhoux F. Role of adipose tissue macrophages in obesity-related disorders. J Exp Med 2022;219:1. 10.1084/jem.20211948 PMC9098652 35543703

[71] Hotamisligil GS, Shargill NS, Spiegelman BM. Adipose expression of tumor necrosis factor-alpha: direct role in obesity-linked insulin resistance. Science 1993;259:87–91. 7678183 10.1126/science.7678183

[72] Rohm TV, Meier DT, Olefsky JM, et al. Inflammation in obesity, diabetes, and related disorders. Immunity 2022b;55:31–55. 35021057 10.1016/j.immuni.2021.12.013 PMC8773457

[73] Castoldi A, Naffah de Souza C, Camara NO, et al. The macrophage switch in obesity development. Front Immunol 2015;6:637. 26779183 10.3389/fimmu.2015.00637 PMC4700258

[74] Lumeng CN, Bodzin JL, Saltiel AR. Obesity induces a phenotypic switch in adipose tissue macrophage polarization. J Clin Invest 2007a;117:175–84. 17200717 10.1172/JCI29881 PMC1716210

[75] Lumeng CN, Deyoung SM, Bodzin JL, et al. Increased inflammatory properties of adipose tissue macrophages recruited during diet-induced obesity. Diabetes 2007b;56:16–23. 17192460 10.2337/db06-1076

[76] Chawla A, Nguyen KD, Goh YP. Macrophage-mediated inflammation in metabolic disease. Nat Rev Immunol 2011;11:738–49. 21984069 10.1038/nri3071 PMC3383854

[77] Zhu L, Yang T, Li L, et al. TSC1 controls macrophage polarization to prevent inflammatory disease. Nat Commun 2014;5:4696. 25175012 10.1038/ncomms5696

[78] Byles V, Covarrubias AJ, Ben-Sahra I, et al. The TSC-mTOR pathway regulates macrophage polarization. Nat Commun 2013;4:2834. 24280772 10.1038/ncomms3834 PMC3876736

[79] Jiang H, Westerterp M, Wang C, et al. Macrophage mTORC1 disruption reduces inflammation and insulin resistance in obese mice. Diabetologia 2014;57:2393–404. 25120095 10.1007/s00125-014-3350-5

[80] Suzuki T, Gao J, Ishigaki Y, et al. ER stress protein CHOP mediates insulin resistance by modulating adipose tissue macrophage polarity. Cell Rep 2017;18:2045–57. 28228268 10.1016/j.celrep.2017.01.076

[81] Shan B, Wang X, Wu Y, et al. The metabolic ER stress sensor IRE1alpha suppresses alternative activation of macrophages and impairs energy expenditure in obesity. Nat Immunol 2017;18:519–29. 28346409 10.1038/ni.3709

[82] Ying W, Fu W, Lee YS, et al. The role of macrophages in obesity-associated islet inflammation and beta-cell abnormalities. Nat Rev Endocrinol 2020;16:81–90. 31836875 10.1038/s41574-019-0286-3 PMC8315273

[83] Zinselmeyer BH, Vomund AN, Saunders BT, et al. The resident macrophages in murine pancreatic islets are constantly probing their local environment, capturing beta cell granules and blood particles. Diabetologia 2018;61:1374–83. 29589072 10.1007/s00125-018-4592-4 PMC5938291

[84] Dalmas E, Lehmann FM, Dror E, et al. Interleukin-33-activated islet-resident innate lymphoid cells promote insulin secretion through myeloid cell retinoic acid production. Immunity 2017;47:928–42.e7 e927. 29166590 10.1016/j.immuni.2017.10.015

[85] Orliaguet L, Dalmas E, Drareni K, et al. Mechanisms of macrophage polarization in insulin signaling and sensitivity. Front Endocrinol (Lausanne) 2020;11:62. 32140136 10.3389/fendo.2020.00062 PMC7042402

[86] Yin Y, Hao H, Cheng Y, et al. Human umbilical cord-derived mesenchymal stem cells direct macrophage polarization to alleviate pancreatic islets dysfunction in type 2 diabetic mice. Cell Death Dis 2018;9:760. 29988034 10.1038/s41419-018-0801-9 PMC6037817

[87] Chittezhath M, Gunaseelan D, Zheng X, et al. Islet macrophages are associated with islet vascular remodeling and compensatory hyperinsulinemia during diabetes. Am J Physiol Endocrinol Metab 2019;317:E1108–20. 31573842 10.1152/ajpendo.00248.2019

[88] Huby T, Gautier EL. Immune cell-mediated features of non-alcoholic steatohepatitis. Nat Rev Immunol 2022;22:429–43. 34741169 10.1038/s41577-021-00639-3 PMC8570243

[89] Wong RJ, Aguilar M, Cheung R, et al. Nonalcoholic steatohepatitis is the second leading etiology of liver disease among adults awaiting liver transplantation in the United States. Gastroenterology 2015;148:547–55. 25461851 10.1053/j.gastro.2014.11.039

[90] Kazankov K, Jorgensen SMD, Thomsen KL, et al. The role of macrophages in nonalcoholic fatty liver disease and nonalcoholic steatohepatitis. Nat Rev Gastroenterol Hepatol 2019;16:145–59. 30482910 10.1038/s41575-018-0082-x

[91] Xu HE, Guo JS. All about NASH: disease biology, targets, and opportunities on the road to NASH drugs. Acta Pharmacol Sin 2022;43:1101–2. 35379932 10.1038/s41401-022-00900-y PMC9061727

[92] Parthasarathy G, Revelo X, Malhi H. Pathogenesis of nonalcoholic steatohepatitis: an overview. Hepatol Commun 2020;4:478–92. 32258944 10.1002/hep4.1479 PMC7109346

[93] Guillot A, Tacke F. Liver macrophages: old dogmas and new insights. Hepatol Commun 2019;3:730–43. 31168508 10.1002/hep4.1356 PMC6545867

[94] Reid DT, Reyes JL, McDonald BA, et al. Kupffer cells undergo fundamental changes during the development of experimental NASH and are critical in initiating liver damage and inflammation. PLoS One 2016;11:e0159524. 27454866 10.1371/journal.pone.0159524 PMC4959686

[95] Koyama Y, Brenner DA. Liver inflammation and fibrosis. J Clin Invest 2017;127:55–64. 28045404 10.1172/JCI88881 PMC5199698

[96] Huang W, Metlakunta A, Dedousis N, et al. Depletion of liver Kupffer cells prevents the development of diet-induced hepatic steatosis and insulin resistance. Diabetes 2010;59:347–357. 19934001 10.2337/db09-0016 PMC2809951

[97] An P, Wei LL, Zhao S, et al. Hepatocyte mitochondria-derived danger signals directly activate hepatic stellate cells and drive progression of liver fibrosis. Nat Commun 2020;11:2362. 32398673 10.1038/s41467-020-16092-0 PMC7217909

[98] Arrese M, Cabrera D, Kalergis AM, et al. Innate immunity and inflammation in NAFLD/NASH. Dig Dis Sci 2016;61:1294–303. 26841783 10.1007/s10620-016-4049-x PMC4948286

[99] Krysko DV, Agostinis P, Krysko O, et al. Emerging role of damage-associated molecular patterns derived from mitochondria in inflammation. Trends Immunol 2011;32:157–64. 21334975 10.1016/j.it.2011.01.005

[100] Mihm S. Danger-associated molecular patterns (DAMPs): molecular triggers for sterile inflammation in the liver. Int J Mol Sci 2018;19:1. 10.3390/ijms19103104 PMC6213769 30309020

[101] Idrissova L, Malhi H, Werneburg NW, et al. TRAIL receptor deletion in mice suppresses the inflammation of nutrient excess. J Hepatol 2015;62:1156–63. 25445398 10.1016/j.jhep.2014.11.033 PMC4404200

[102] Soltis AR, Kennedy NJ, Xin X, et al. Hepatic dysfunction caused by consumption of a high-fat diet. Cell Rep 2017;21:3317–28. 29241556 10.1016/j.celrep.2017.11.059 PMC5734865

[103] Galluzzi L, Vitale I, Aaronson SA, et al. Molecular mechanisms of cell death: recommendations of the Nomenclature Committee on Cell Death 2018. Cell Death Differ 2018;25:486–541. 29362479 10.1038/s41418-017-0012-4 PMC5864239

[104] Gautheron J, Vucur M, Luedde T. Necroptosis in nonalcoholic steatohepatitis. Cell Mol Gastroenterol Hepatol 2015;1:264–5. 28210679 10.1016/j.jcmgh.2015.02.001 PMC5301189

[105] Xu B, Jiang M, Chu Y, et al. Gasdermin D plays a key role as a pyroptosis executor of non-alcoholic steatohepatitis in humans and mice. J Hepatol 2018;68:773–82. 29273476 10.1016/j.jhep.2017.11.040

[106] Chen S, Zhu JY, Zang X, et al. The emerging role of ferroptosis in liver diseases. Front Cell Dev Biol 2021;9:801365. 34970553 10.3389/fcell.2021.801365 PMC8713249

[107] Qi J, Kim JW, Zhou Z, et al. Ferroptosis affects the progression of nonalcoholic steatohepatitis via the modulation of lipid peroxidation-mediated cell death in mice. Am J Pathol 2020;190:68–81. 31610178 10.1016/j.ajpath.2019.09.011

[108] Tsurusaki S, Tsuchiya Y, Koumura T, et al. Hepatic ferroptosis plays an important role as the trigger for initiating inflammation in nonalcoholic steatohepatitis. Cell Death Dis 2019;10:449. 31209199 10.1038/s41419-019-1678-y PMC6579767

[109] Schwabe RF, Luedde T. Apoptosis and necroptosis in the liver: a matter of life and death. Nat Rev Gastroenterol Hepatol 2018;15:738–52. 30250076 10.1038/s41575-018-0065-y PMC6490680

[110] Shojaie L, Iorga A, Dara L. Cell death in liver diseases: a review. Int J Mol Sci 2020;21:1. 10.3390/ijms21249682 PMC7766597 33353156

[111] Wan J, Weiss E, Ben Mkaddem S, et al. LC3-associated phagocytosis protects against inflammation and liver fibrosis via immunoreceptor inhibitory signaling. Sci Transl Med 2020;12:1. 10.1126/scitranslmed.aaw8523 32295902

[112] Gordon S. Phagocytosis: an immunobiologic process. Immunity 2016a;44:463–75. 26982354 10.1016/j.immuni.2016.02.026

[113] Varol C, Mildner A, Jung S. Macrophages: development and tissue specialization. Annu Rev Immunol 2015;33:643–75. 25861979 10.1146/annurev-immunol-032414-112220

[114] Inoue Y, Fukui H, Xu X, et al. Colonic M1 macrophage is associated with the prolongation of gastrointestinal motility and obesity in mice treated with vancomycin. Mol Med Rep 2019;19:2591–8. 30720127 10.3892/mmr.2019.9920 PMC6423659

[115] Srivastava A, Chau K, Kwon H, et al. Early and frequent exposure to antibiotics in children and the risk of obesity: systematic review and meta-analysis of observational studies. F1000Res 2020;9:711. 32913641 10.12688/f1000research.24553.1 PMC7429923

[116] Vallianou N, Dalamaga M, Stratigou T, et al. Do antibiotics cause obesity through long-term alterations in the gut microbiome? A review of current evidence. Curr Obes Rep 2021;10:244–62. 33945146 10.1007/s13679-021-00438-w PMC8093917

[117] Scott NA, Andrusaite A, Andersen P, et al. Antibiotics induce sustained dysregulation of intestinal T cell immunity by perturbing macrophage homeostasis. Sci Transl Med 2018;10:1. 10.1126/scitranslmed.aao4755 PMC6548564 30355800

[118] Rohm TV, Fuchs R, Muller RL, et al. Obesity in humans is characterized by gut inflammation as shown by pro-inflammatory intestinal macrophage accumulation. Front Immunol 2021;12:668654. 34054838 10.3389/fimmu.2021.668654 PMC8158297

[119] Biswas SK, Bonecchi R. Colonic macrophages “remote control” adipose tissue inflammation and insulin resistance. Cell Metab 2016;24:196–8. 27508866 10.1016/j.cmet.2016.07.020

[120] Rohm TV, Keller L, Bosch AJT, et al. Targeting colonic macrophages improves glycemic control in high-fat diet-induced obesity. Commun Biol 2022a;5:370. 35440795 10.1038/s42003-022-03305-z PMC9018739

[121] Scheithauer TPM, Rampanelli E, Nieuwdorp M, et al. Gut microbiota as a trigger for metabolic inflammation in obesity and type 2 diabetes. Front Immunol 2020;11:571731. 33178196 10.3389/fimmu.2020.571731 PMC7596417

[122] Winer DA, Winer S, Dranse HJ, et al. Immunologic impact of the intestine in metabolic disease. J Clin Invest 2017;127:33–42. 28045403 10.1172/JCI88879 PMC5199708

[123] Rooks MG, Garrett WS. Gut microbiota, metabolites and host immunity. Nat Rev Immunol 2016;16:341–52. 27231050 10.1038/nri.2016.42 PMC5541232

[124] Zheng D, Liwinski T, Elinav E. Interaction between microbiota and immunity in health and disease. Cell Res 2020;30:492–506. 32433595 10.1038/s41422-020-0332-7 PMC7264227

[125] Turnbaugh PJ, Ridaura VK, Faith JJ, et al. The effect of diet on the human gut microbiome: a metagenomic analysis in humanized gnotobiotic mice. Sci Transl Med 2009;1:6–ra14. 10.1126/scitranslmed.3000322 PMC2894525 20368178

[126] Vrieze A, Van Nood E, Holleman F, et al. Transfer of intestinal microbiota from lean donors increases insulin sensitivity in individuals with metabolic syndrome. Gastroenterology 2012;143:913–6 e917. 22728514 10.1053/j.gastro.2012.06.031

[127] Wu T, Li H, Su C, et al. Microbiota-derived short-chain fatty acids promote LAMTOR2-mediated immune responses in macrophages. mSystems 2020;5:e00587-20. 10.1128/mSystems.00587-20 PMC7646525 33144310

[128] Nakamura A, Kurihara S, Takahashi D, et al. Symbiotic polyamine metabolism regulates epithelial proliferation and macrophage differentiation in the colon. Nat Commun 2021;12:2105. 33833232 10.1038/s41467-021-22212-1 PMC8032791

[129] Su CW, Chen CY, Li Y, et al. Helminth infection protects against high fat diet-induced obesity via induction of alternatively activated macrophages. Sci Rep 2018;8:4607. 29545532 10.1038/s41598-018-22920-7 PMC5854586

[130] Rapin A, Chuat A, Lebon L, et al. Infection with a small intestinal helminth, Heligmosomoides polygyrus bakeri, consistently alters microbial communities throughout the murine small and large intestine. Int J Parasitol 2020;50:35–46. 31759944 10.1016/j.ijpara.2019.09.005

[131] Walk ST, Blum AM, Ewing SA, et al. Alteration of the murine gut microbiota during infection with the parasitic helminth Heligmosomoides polygyrus. Inflamm Bowel Dis 2010;16:1841–9. 20848461 10.1002/ibd.21299 PMC2959136

[132] McCurdy CE, Bishop JM, Williams SM, et al. Maternal high-fat diet triggers lipotoxicity in the fetal livers of nonhuman primates. J Clin Invest 2009;119:323–35. 19147984 10.1172/JCI32661 PMC2631287

[133] Mouralidarane A, Soeda J, Visconti-Pugmire C, et al. Maternal obesity programs offspring nonalcoholic fatty liver disease by innate immune dysfunction in mice. Hepatology 2013;58:128–38. 23315950 10.1002/hep.26248

[134] Oben JA, Mouralidarane A, Samuelsson AM, et al. Maternal obesity during pregnancy and lactation programs the development of offspring non-alcoholic fatty liver disease in mice. J Hepatol 2010;52:913–20. 20413174 10.1016/j.jhep.2009.12.042

[135] Thorn SR, Baquero KC, Newsom SA, et al. Early life exposure to maternal insulin resistance has persistent effects on hepatic NAFLD in juvenile nonhuman primates. Diabetes 2014;63:2702–13. 24705404 10.2337/db14-0276 PMC4113070

[136] Soderborg TK, Clark SE, Mulligan CE, et al. The gut microbiota in infants of obese mothers increases inflammation and susceptibility to NAFLD. Nat Commun 2018;9:4462. 30367045 10.1038/s41467-018-06929-0 PMC6203757

[137] Mathieu M, Martin-Jaular L, Lavieu G, et al. Specificities of secretion and uptake of exosomes and other extracellular vesicles for cell-to-cell communication. Nat Cell Biol 2019;21:9–17. 30602770 10.1038/s41556-018-0250-9

[138] Choi Y, Kwon Y, Kim DK, et al. Gut microbe-derived extracellular vesicles induce insulin resistance, thereby impairing glucose metabolism in skeletal muscle. Sci Rep 2015;5:15878. 26510393 10.1038/srep15878 PMC4625370

[139] Luo Z, Ji Y, Gao H, et al. CRIg(+) macrophages prevent gut microbial DNA-containing extracellular vesicle-induced tissue inflammation and insulin resistance. Gastroenterology 2021;160:863–74. 33152356 10.1053/j.gastro.2020.10.042 PMC7878308

[140] Bai J, Liu F. The cGAS-cGAMP-STING pathway: a molecular link between immunity and metabolism. Diabetes 2019;68:1099–108. 31109939 10.2337/dbi18-0052 PMC6610018

[141] Bai J, Liu F. cGASSTING signaling and function in metabolism and kidney diseases. J Mol Cell Biol 2021;13:728–38. 34665236 10.1093/jmcb/mjab066 PMC8718186

[142] Cai D, Gao W, Li Z, et al. Current development of nano-drug delivery to target macrophages. Biomedicines 2022;10:12031. 10.3390/biomedicines10051203 PMC9139084 35625939

[143] Soares S, Sousa J, Pais A, et al. Nanomedicine: principles, properties, and regulatory issues. Front Chem 2018;6:360. 30177965 10.3389/fchem.2018.00360 PMC6109690

[144] Chen W, Schilperoort M, Cao Y, et al. Macrophage-targeted nanomedicine for the diagnosis and treatment of atherosclerosis. Nat Rev Cardiol 2022c;19:228–49. 34759324 10.1038/s41569-021-00629-x PMC8580169

[145] Lameijer MA, Tang J, Nahrendorf M, et al. Monocytes and macrophages as nanomedicinal targets for improved diagnosis and treatment of disease. Expert Rev Mol Diagn 2013;13:567–80. 23895127 10.1586/14737159.2013.819216 PMC4110962

